# Predicting Everyday Critical Thinking: A Review of Critical Thinking Assessments

**DOI:** 10.3390/jintelligence12020016

**Published:** 2024-02-01

**Authors:** Heather A. Butler

**Affiliations:** Department of Psychology, California State University, Dominguez Hills, Carson, CA 90747, USA; hbutler@csudh.edu

**Keywords:** critical thinking assessment, critical thinking skills, critical thinking disposition, everyday outcomes of thinking, reasoning, logic, cognitive bias

## Abstract

Our ability to think critically and our disposition to do so can have major implications for our everyday lives. Research across the globe has shown the impact of critical thinking on decisions about our health, politics, relationships, finances, consumer purchases, education, work, and more. This chapter will review some of that research. Given the importance of critical thinking to our everyday lives, the fair and unbiased assessment of critical thinking is useful for guiding educators in their classrooms, for the sake of self-improvement, and in employment decisions. This chapter will also review the psychometric properties of several critical thinking assessments, with a special emphasis on the everyday behaviors predicted by these assessments. The practical challenges faced by test adopters and future directions in the assessment of critical thinking will be discussed.

## 1. Introduction

In 2022, a poll conducted by the Pearson Institute for the Study of Resolution of Global Conflicts revealed that 91% of citizens of the United States believed misinformation was a significant problem (Klepper 2022); the same poll found that only 44% of them believed they had been involved in spreading misinformation. It seems that people recognized that there was a problem but did not believe they were contributing to the problem. When asked who was to blame for the spread of misinformation respondents identified the government (72% identified U.S, politicians, 48% the U.S. government, 54% Russia, 53% China, 39% Iran, 41% other foreign governments) and social media (77% identified social media users, 73% social media companies) as the main culprits. It is wise for respondents to be concerned about the spread of misinformation on social media; it has become a major source of news and is largely unregulated. A 2022 survey conducted by the Pew Research Institute (Liedke and Wang 2023), found that more than half of the adults in the United States regularly get their news from social media sites such as Facebook (31%), YouTube (25%), Twitter (14%), Instagram (13%), TikTok (10%), and others. Many scholars have voiced their concerns about the growing use of social media sites as a source of information due to concerns about echo chambers and the ease with which misinformation can be spread (Bakshy et al. 2015).

It is estimated that people across the world spend an average of 2.5 h per day on social media (Ali and AJLabs 2023), but the information consumed during that time is not a balanced representation of all viewpoints. All mainstream social media websites use algorithms that push content to you based on your usage (e.g., videos you have watched completely or repeatedly, posts you have interacted with by sharing or liking them). These algorithms learn enough about you that they begin to feed you information that is consistent with your interests or preexisting beliefs. For example, at the time this chapter was written, the author was pregnant with her first child and the algorithms fed her content about pregnancy, labor, and parenting. The algorithms were so savvy that they even triangulated which trimester she was in and fed her content accordingly. Some of this content was produced by reputable sources who were experts in their fields and cited credible sources (e.g., a doctor who is board-certified in fetal medicine citing quality empirical research), but most of it was not (e.g., medical advice being given by a chiropractor that was not based on any research), and some of the questionable content directly contradicted the evidence-based medical advice given by the reputable doctor while insinuating that the credible information should not be trusted. It was alarming to see bad medical advice being given so freely by these content creators, but it was also clear that many consumers of this content were uncritically accepting the information based on their comments and were likely exposed to a lot of similar content.

The danger associated with these social media algorithms is that they create echo chambers that insulate us from different perspectives and feed us information that is already consistent with our existing beliefs (Bakshy et al. 2015), thereby strengthening the conviction of our existing beliefs and potentially inflating our perception of how many others share that belief. This does not encourage critical thinking. The more videos you see of people espousing similar beliefs to you, the more you come to believe that most people believe the same thing that you believe. You are less likely to be exposed to, and consider, alternative viewpoints, and are more likely to commit confirmation bias (the tendency to seek out, and eagerness to accept, information that is consistent with your preexisting beliefs). Imagine a person who is distrustful of science: when they see social media content that is critical of science or medicine, they “like” the post. The algorithms then feed them more content that is critical of medicine and soon much of the content they consume is stories about medical mistakes, negative experiences with doctors, and positive experiences with more holistic practices. They read the comments of others interacting with this content and most seem to agree that the medical establishment should not be trusted, so they conclude that doctors are dangerous and that most people share this belief. The rise in a distrust of science has been well documented (Tsipursky 2018), but it is certainly not the only domain in our lives impacted by these echo chambers. Echo chambers have been implicated for their role in the rise of partisan politics in the United States (Frenkel and Isaac 2018) and can contribute to the phenomenon known as *the group polarization effect* (the tendency for the views of like-minded people to become more extreme when they discuss their opinions on the topics they share similar beliefs about).

More recently, artificial intelligence (AI) has contributed to the spread of misinformation by generating fake images and videos. Creators of this content could technically be accused of spreading *dis*information, since the intent of the sharer of such information is to mislead consumers. The problem has become so widespread that *Rolling Stone* magazine published a story on the problem, urging readers to use their good judgement before sharing stories online (Klee and McCann Ramirez 2023). The article blamed AI technology for fanning political flames and spreading misinformation about the Israel–Hamas conflict. It is unfortunate that the rise of social media usage will likely result in less critical thinking about the information we consume online. The enormous benefits of the Internet are undeniable, but educators across the globe are encouraged to discuss with their students the damaging impact that echo chambers can have on our everyday lives by making critical thinking more difficult.

## 2. How Critical Thinking Impacts Everyday Life

The ability to think critically does not guarantee us a good life that is free from bias or errors, and it does not guarantee that we will not fall prey to bad advice given on social media, but it may protect us from experiencing certain negative life events. In a series of studies, researchers measured the extent to which critical thinking predicted the occurrence of certain everyday life outcomes (Butler 2012; Butler et al. 2012, 2017). Community adults from several countries took a well-established critical thinking assessment (the Halpern Critical Thinking Assessment) and completed an inventory of negative life events. The inventory of negative life events was adapted from a decision-making competence inventory (Bruine de Bruin et al. 2007). The inventory was unique in that it allowed the researchers to measure the *proportion* of negative life events experienced by the respondent by inventorying both the negative life event and the neutral life event that may have made the negative event possible. For instance, the respondents were asked whether they had driven a car (a neutral life event) and then whether they had ever been arrested for driving under the influence of drugs or alcohol (a negative life event also known as a DUI in the United States). If you only asked whether the respondent had received a DUI and the respondent reported that they did not get a DUI, you would not know whether they did not get a DUI because they made the good decision not to drive under the influence of drugs or alcohol or whether they did not drive a car at all because they do not have a license to drive. Thus, this unique inventory allowed researchers to measure the proportion of negative life events experienced by the respondents. The everyday life events ranged in severity from trivial (e.g., I ruined a load a laundry) to severe (e.g., I contracted a sexually transmitted disease by failing to use a condom when I had sex). They also measured experiences across various domains of life, such as health (e.g., I had or was responsible for an unplanned pregnancy), safety (e.g., I was arrested for driving under the influence of drugs or alcohol), finances (e.g., I was charged a late fee because I did not pay my bill on time), social/interpersonal (e.g., I cheated on my significant other of more than one year), and education (e.g., I forgot about a scheduled exam). The researchers found that those who scored higher on the critical thinking assessment experienced fewer negative life events, compared to those who scored lower on the critical thinking assessment. The authors concluded that thinking critically offers us some protection from making questionable life decisions. Another benefit of the inventory used in this research is that it captured self-reported behaviors, which offers some insight into the respondents’ dispositions towards making good decisions and thinking critically.

## 3. Critical Thinking: Skills and Dispositions

The disposition to use one’s critical thinking skills is as important as the skills themselves. If a person understands the skills involved in thinking critically but fails to deploy those skills when the situation warrants, they would not be classified as a critical thinker. Imagine a person who understands that causation should not be inferred from correlational research but accepts as truth medical advice based on correlation. This is what happened with a common vaccine given in childhood. Despite several large-scale studies confirming that the Measles, Mumps, and Rubella (MMR) vaccine was not responsible for causing autism (see Jain et al. 2015) parents in several countries elected not to give the vaccine to their children and this had severe consequences. Europe saw a 400% increase in measles from 2016–2017 (World Health Organization 2018). In 2015, 10% of children in the United States were not vaccinated for the disease, which had nearly been irradicated (National Center for Health Statistics 2015, tab. 67). In Romania, celebrities took to social media to warn parents not to vaccinate their children and to drink cabbage juice instead. Dozens of infants died due to a major outbreak (Gheorghia 2018). Correlational research found that autism was diagnosed around the same time a vaccine was given to children and incorrectly concluded that the vaccine was causing autism. Decades later, we still do not know what causes autism, but we do know that it is not the vaccine. Yet, hundreds of well-meaning parents question whether to give their child the vaccine each year.

Psychologists and philosophers have debated the exact definition of critical thinking for decades, as well as whether the construct is domain-specific or domain-general, but most definitions of critical thinking include thinking that is logical and free of bias. In her book, *Thought and Knowledge*, Halpern defined critical thinking as:

“the use of those cognitive skills and abilities that increase the probability of a desirable outcome. It is used to describe thinking that is purposeful, reasoned, and goal directed—the kind of thinking involved in solving problems, formulating inferences, calculating likelihoods, and making decisions”

(Halpern 2014, p. 8).

Critical thinking also differs from intelligence, although both constructs refer to cognitive abilities. Stanovich and West (2008) and others have argued that our everyday definition of intelligence more accurately describes critical thinking than what most intelligence tests measure, which tends to be short-term memory, vocabulary, analogies, and spatial skills (Butler and Halpern 2020). In terms of predicting behavior, both critical thinking and intelligence can predict everyday behavior. Butler et al. (2017) compared the predictive power of an intelligence test to the predictive power of a critical thinking assessment. Participants took the INSBAT intelligence test (Arendasy et al. 2012), the Halpern Critical Thinking Assessment (HCTA; Halpern 2012), and the real-world outcomes inventory (the same inventory discussed previously; Butler 2012). Overall, both predicted negative life events, although the critical thinking assessment did a slightly better job at this than the intelligence test. That is, those who scored high on the intelligence test and those who scored high on the critical thinking assessment reported experiencing fewer negative life events. Interestingly, the critical thinking scores accounted for unique variance in the model beyond the variance intelligence scores accounted for. This implies that the constructs of intelligence and critical thinking are different, but more importantly that they are impacting our everyday lives in different ways.

One of the advantages of critical thinking over intelligence is that it is easier to teach someone to be a critical thinker than it is to improve their intelligence. Each year, thousands of college students enroll in critical thinking courses and nearly every university includes critical thinking as a university-wide student learning outcome. Despite this, there is evidence that the critical thinking abilities of over one-third of college students do not improve during their time in college. The book, *Academically Adrift* (Arum and Roksa 2010), discusses this finding in an analysis that is critical of higher education. The book has been criticized for being overly pessimistic and ignoring the lack of incentives offered to students who participated in the research. The publication was successful in prompting a thorough meta-analysis of the topic, which reached a different conclusion about whether the academy was successful in training critical thinkers. Huber and Kuncel (2015) analyzed 71 studies conducted over a 48 year period that measured changes in the critical thinking of college students during their time in the academy. The meta-analysis concluded that critical thinking skills and critical thinking dispositions improved over the college experience. That said, the gains in critical thinking became smaller as time passed, indicating that students are not learning thinking skills and developing a disposition towards critical thinking as much as they have in the past. The authors argue that the findings could be the result of changes to the college curriculum, changes in student behavior, and the increase in critical thinking instruction while students are still in high school, middle, or elementary school.

If we truly value critical thinking in education, then we should be measuring whether our students are learning to think critically in our classrooms. Colleges and universities would do well to provide resources for these important assessment efforts.

## 4. Measuring Critical Thinking

### 4.1. Practical Challenges

There are many practical challenges to the assessment of critical thinking, especially on college campuses. First, there is little incentive to do so. While many colleges and universities declare critical thinking as an important learning outcome of the education they provide, few require high-quality evidence that it is occurring. Much of the assessment work done at a university-wide level is done during accreditation by a few faculty members under the guise of service to the university. It is not embedded in normal university activities and is viewed as additional work, which makes it more likely that the easiest path of assessment is chosen over the most accurate. Furthermore, the details of assessment work are rarely shared broadly with the entire university or discussed as a responsibility of all faculty. Second, it takes time to assess critical thinking well. There are several different types of assessment to choose from. It takes time to select one (and knowledge of what to look for in a quality assessment). One of the most fundamental differences between these assessments is whether the questions are multiple-choice or forced-choice questions that rely on recognition memory or short-answer questions that rely on recall memory. The weakness of a forced-choice question is that respondents are being provided a memory cue that may make it easier to guess the correct answer, which is less of a concern with a short-answer question that does not provide any cues. A challenge of the short-answer question is that the answers will be more difficult to grade and may be more susceptible to biased grading. Third, most critical thinking assessments cost money. Colleges and universities must provide the financial resources to purchase the assessments and to have them graded. Despite the practical challenges of critical thinking assessment, we believe it to be an important endeavor that universities should prioritize. It should also be a recursive process, whereby the information gained from the assessment is shared with educators who can then use it to improve instruction, which is then visible in subsequent assessments.

Regardless of setting, the assessment of complex constructs is challenging, and it is especially difficult to measure a complex construct like critical thinking when the definition of critical thinking is still debated by scholars. How a test developer defines critical thinking plays a role in how it is measured and the factors that are included. As you will see from the review of several critical thinking assessments below, while each assessment provides an overall score for critical thinking skills or dispositions, the subscales used to create this overall score differ based on how the construct was defined by the test developer. Some test developers adopted a more conceptual approach (e.g., using the Delphi Report’s definition of critical thinking to guide the test’s development), while others were guided by the psychometric properties of their assessments. While differences exist both in the definition of critical thinking and the skills that developers choose to include, most critical thinking skill assessments measure some form of argument analysis, questioning assumptions, inductive and deductive reasoning, and quantitative reasoning.

One area where critical thinking assessment has done particularly well has been the emphasis on realistic assessment scenarios. Many of the assessments reviewed in this chapter ask respondents to respond to everyday scenarios, such as evaluating a letter to the editor of a newspaper, or a statement made by a politician. These scenarios are the very everyday life situations we hope respondents are using their critical thinking skills to evaluate. Unfortunately, many critical thinking assessments fail to confirm that performance on the assessment predicts everyday behavior. Most assessments of critical thinking use academic performance to demonstrate the predictive (criterion) validity of the assessment. If your goal is to assess whether learners are applying critical thinking skills in the classroom only, then perhaps this gap in predictive power may not seem particularly troublesome. If your goal is to assess whether the knowledge gained by learning thinking skills transfers to other domains of life for the betterment of the individual and society, then this is an area where many critical thinking assessments fall short. As you will see in the review that follows, only a few assessments have demonstrated that scores on their assessments predict everyday behavior.

### 4.2. Critical Thinking Assessments

This section will examine the psychometric qualities of eight critical thinking assessments: six assessments measure cognitive skills associated with critical thinking and two measure critical thinking dispositions. This is not an exhaustive list of critical thinking assessments. Six assessments utilize a multiple-choice (recognition memory) format only: the California Critical Thinking Dispositions Inventory (CCTDI), California Critical Thinking Skills Test (CCTST), Cornell Critical Thinking Test (CCTT), the California Measure of Mental Motivation (CM3), the Test of Everyday Reasoning (TER), and the Watson–Glaser^TM^ II Critical Thinking Appraisal (W-GII). One assessment relies exclusively on a short-answer (recall memory) format, the Ennis–Weir Critical Thinking Essay. Only one assessment utilizes both multiple-choice and short-answer, the Halpern Critical Thinking Assessment (HCTA). For a concise list of assessment attributes, see Table 1.

#### 4.2.1. California Critical Thinking Dispositions Inventory (CCTDI; Insight Assessment, Inc. n.d.)

Insight Assessment is the developer of this assessment, which was originally authored by Facione (1990) to measure an individual’s tendency to think critically. The assessment measures truth-seeking, open-mindedness, analyticity, systematicity, critical thinking confidence, inquisitiveness, and maturity of judgment. It is intended for use with undergraduate and graduate students. The assessment contains 75 items and takes 30 min to complete. The CCTDI asks respondents the extent to which they agree or disagree with a series of questions. For example, respondents might be asked whether “it is important to me to figure out what people really mean by what they say” or “changing your mind is a sign of weakness” (reverse scored). It is available in both digital and paper form in multiple languages, including English, French, Spanish, Chinese, Japanese, and 14 others. To administer this assessment, you must have the appropriate credentials and formal training in administering and scoring clinical assessments ethically.

The seven factors measured by this assessment are based on the Delphi Report’s definition of critical thinking. Subsequent research conducted by Walsh et al. (2007) did not support the seven-factor structure and instead recommended a four-factor structure, but the test is still being advertised as measuring the seven original factors. The internal reliability of the CCTDI is good (Cronbach α = 0.91) but varies based on the type of sample (e.g., nursing students, college students).

#### 4.2.2. California Critical Thinking Skills Test (CCTST; Insight Assessment, Inc. n.d.)

The developers of this assessment state that it is the most widely used critical thinking assessment in the world. It measures problem analysis, interpretation, inference, evaluation of arguments, explanation (providing evidence, assumptions, and rational decision-making), induction, deduction, and numeracy (quantitative reasoning). It is intended for use with college undergraduate and graduate students. The assessment contains 40 scenarios that test-takers respond to by selecting a given response. It is available online in multiple languages including English, Arabic, Chinese Simplified, Chinese Traditional, Dutch, French, German, Indonesian-Bahasa, Italian, Japanese, Korean, Norwegian, Portuguese, Spanish, Swedish, Thai, Turkish, and Vietnamese. No specific license is required to administer this assessment, but it is only sold to educational institutions, educational consultations, or other educationally related organizations such as the Department of Education or the National Science Foundation.

The manual for this assessment cites publications that provide evidence of reliability and validity. It was validated with college students (community college, undergraduate, graduate, law, and MBA), employees, military personnel, children K-12, health professionals, and the general population. It was also tested against the influence of social desirability and culture bias. In terms of content validity, only face validity was provided; namely, that the factors measured by this assessment were based on the Delphi Report’s definition of critical thinking. There is evidence supporting the construct validity of the assessment. The strongest evidence compared scores on the assessment to scores on the GRE (GRE Total Score *r* = 0.719, GRE Analytic *r* = 0.708, GRE Verbal *r* = 0.716, GRE Quantitative, *r* = 0.582). The relationship between academic performance and scores on the assessment was weak to moderate (ranging from 0.20 to 0.46), but the developers argue that more goes into grades than just a student’s ability to think such as participation and content knowledge. In terms of criterion validity, the assessment has been used to evaluate training programs, learning outcomes in educational settings, and decision-making in employment settings. These evaluations occurred largely with medical and nursing students. The internal consistency of the measure is sufficient (e.g., most tests exceeded the minimum standard 0.70), as is the test–retest reliability (0.80). The factor loadings for the items ranged from 0.30 to 0.77, indicating a questionable factor structure, as was the case with the CCTDI.

#### 4.2.3. Cornell Critical Thinking Test (CCTT; The Critical Thinking Company n.d.)

This assessment measures critical thinking skills and abilities. There are two versions of the assessment: level X was developed for use with students grades 5 to 12 and level Z was developed for use with students in grade 11 to adulthood. Level X advertises that it measures induction, deduction, credibility, and the identification of assumption. It consists of 71 items and takes 50 min to complete. Level Z advertises that it measures induction, deduction, credibility, semantics, definition, prediction and planning experiments, and the identification of assumption. It consists of 52 items and takes 50 min to complete. Both versions of the assessment rely on recognition memory (multiple-choice items). Neither assessment is available online; only a paper version is available. It is available in English. There is a fee for this assessment, but no credentials are required to administer it.

According to the publisher of the assessment (The Critical Thinking Company n.d.) evidence of the assessment’s reliability and validity can be found in the manual, which was not available publicly at the time this chapter was written. There have been a few published and peer-reviewed studies of the assessment that provide weak evidence to support its reliability and validity. In terms of the factor structure, Michael et al. (1980) did not find evidence to support the measurement of the factors proposed by the test developer (only one factor corresponded to that of the developer) and French et al. (2012) found that 94% of the items were potentially biased and showed differential item functioning based on gender. In terms of reliability, the evidence varied, but none met the recommended standards for reliability. The internal consistency of the tests ranged from 0.52 to 0.77 and split-half reliability ranged from 0.55 to 0.76 (Bart 2010). In terms of validity, the relationship between scores on the assessment and student grades was rather weak (*r* = 0.15–0.17; Michael et al. 1980); the relationship with standardized language or quantitative reasoning was modest (0.51–0.62; Landis and Michael 1981); and the relationship with scholastic aptitude and intelligence measures were strong (approximately 0.50 for both). In 2005, following the publication of the research evaluating the psychometric qualities of this assessment, the assessment was refined. Unfortunately, the research establishing the measure as reliable and valid was not available publicly at the time of this chapter’s publication. It is available in the manual provided upon purchase.

#### 4.2.4. California Measure of Mental Motivation (CM3; Insight Assessment, Inc. n.d.)

Insight Assessment is the developer of this assessment, which measures cognitive engagement and motivation towards problem solving and learning in children and adolescents (K-12+). It is available in both digital and paper versions, and in multiple languages including English, Chinese, Spanish, Arabic, and Greek (Insight Assessment, Inc. n.d.). Several versions are available based on the age of the respondent. The assessment contains approximately 25 items (it varies based on the version) and takes approximately 20 min to complete. There is a fee for this assessment. Confirmatory factor analysis on the 25-item instrument found four distinct constructs that ranged in internal consistency from 0.73 to 0.87 (Giancarlo et al. 2004). The four constructs were learning orientation, creative problem solving, mental focus, and cognitive integrity. The criterion validity of the assessment was assessed by comparing scores on the assessment to scores on measures of self-efficacy (*r* = 0.28) and academic achievement, including scores on the SAT (*r* = 0.10 to 0.46) and GPA (*r* = 0.19 to 0.46).

#### 4.2.5. Ennis–Weir Critical Thinking Essay Test (Ennis and Weir 2005)

This assessment measures critical thinking (primarily argumentation and evaluation) by asking respondents to evaluate fictitious letters to newspaper editors. It was intended as a teaching tool, to be used as a framework a short critical thinking course or to be embedded as an assessment tool within a full critical thinking course. The psychometric qualities of the assessment have been extensively studied in 24 studies (Ennis 2005; Ennis and Weir 2005). Bart (2010) found that both the external validity and the content validity of the assessment were good, but criterion validity has not been established. In terms of reliability, the interrater reliability of the assessment is acceptable (*r* = 0.86 to 0.99 for the college student sample), but the internal reliability of the assessment was not acceptable (Cronbach’s α = 0.59 for the college student sample). The lack of internal reliability and criterion validity associated with this assessment makes its use questionable. That said, it was the only assessment we reviewed that was free, which may appeal to resource-strained educators who intend to use it for its intended purpose as a tool in the classroom.

#### 4.2.6. Halpern Critical Thinking Assessment (HCTA; Halpern 2012)

This assessment measures verbal reasoning, argument analysis, hypothesis testing, likelihoods, and decision-making/problem-solving. It was available for a fee through the Vienna Test System (www.schuhfried.com accessed on 1 May 2018) for a time but has since been retired. The target audience for the assessment was adults. Both versions of the assessment included 20 scenarios drawn from different aspects of everyday life. The short version of the assessment took 20 min to complete and included multiple-choice response options only, while the longer version of the assessment took 45 min to complete and included both the multiple-choice questions and short-answer questions. The assessment included computer-assisted grading of the written responses, which guided novice grades through grading the assessment.

There is research confirming the reliability and validity of this assessment (see Halpern 2012). In terms of reliability, both the internal consistency of the assessment (Cronbach’s α = 0.88) and the interrater reliability (*r* = 0.93) are strong. It should be noted that the interrater reliability was established with the computerized grading system, which guides graders through the processing of grading the short-answer responses. In terms of validity, construct and criterion validity have been established. The factor structure was confirmed in two studies. Numerous studies have evaluated the construct validity of the assessment with samples from different countries. The relationship between responses to the multiple-choice questions and responses to the short-answer questions were examined in four separate studies and indicate the two versions of the assessment measure separate, but related factors (*r* = 0.39 to 0.51). The criterion validity of the assessment was established by comparing scores on the assessment to students’ GPA (*r* = 0.35) and standardized exam scores (SAT-Verbal *r* = 0.58, SAT-Math *r* = 0.50, GRE-Verbal *r* = 0.12, GRE-Quantitative *r* = 0.20). Scores on the assessment have also been compared to scores on a personality assessment measuring conscientiousness (*r* = 0.02), the Arlin Test of Formal Reasoning (*r* = 0.32), and scores on the Need for Cognition Scale (*r* = 0.34). And finally, as already discussed, scores on the assessment predicted real-world behaviors such that they were inversely related to the proportion of negative life events experienced by a group of community adults and college students who took the assessment online (Butler 2012; Butler et al. 2012, 2017). This relationship was found in numerous countries (e.g., the United States, Ireland, Portugal). Although this assessment is no longer available, we include it as an example of assessment with excellent psychometric qualities that predicts behavior in everyday life and encourage readers to consider developing similar measures.

#### 4.2.7. Test of Everyday Reasoning (TER; Insight Assessment, Inc. n.d.)

This test is available from Insight Assessment. The developer states that it measures analysis, interpretation, inference, evaluation, explanation, numeracy, deduction, and induction. It is available in both digital and paper formats. The assessment contains 35 items that respondents respond to by selecting one of two options (dichotomous choice). The test is available in English, Greek, Russian, and Spanish. There is a fee for the assessment. In terms of reliability, the internal consistency of the assessment ranged from 0.71 to 0.86 (Facione et al. 2012). No evidence of validity was available for this assessment.

#### 4.2.8. Watson–Glaser^TM^ II Critical Thinking Appraisal (W-GII; NCS Pearson, Inc. 2009)

This assessment measures inference, assumptions, deduction, interpretation, and argument evaluation. The problem-based assessment uses multiple-choice questions with varying numbers of response options. It is marketed to employers but could be used in a variety of settings. It contains 40 questions and takes roughly 30 min to complete. There is a fee for the assessment, which is available in a digital/online format. The developer provides two practice tests, drills, and five interactive study guides on their website. One sample item that measures inference asks respondents to read the passage and “choose whether each of the statements that follow are true or false to varying degrees…”. The scenario is as follows.

“Virtual employees, or employees who work from home via a computer, are an increasing trend. In the US, the number of virtual employees has increased by 39% in the last two years and 74% in the last five years. Employing virtual workers reduces costs and makes it possible to use talented workers no matter where they are located globally. Yet, running a workplace with virtual employees might entail miscommunication and less camaraderie and can be more time-consuming than face-to-face interaction”.

Respondents answer two questions about this passage. The first question is “The marked advantage of virtual employee hiring is the ability to benefit from the output of unsociable employees without involving them in face-to-face interactions” and the second question is “Today, a majority of the employees in the US are virtual employees”. Respondents answer by selecting one option: true, probably true, insufficient data, probably false, or false.

There is research confirming the reliability and validity of this assessment (see NCS Pearson, Inc. 2009). In terms of reliability, research supports both the factor structure and internal consistency of the assessment. A factor analysis revealed three factors: recognizing assumptions, evaluating arguments, and drawing conclusions. The internal consistency of the assessment was good, it ranged from 0.81 to 0.89. Convergent and criterion validity have been established. Tests of convergent validity compared scores on the assessment to scores on several tests of intelligence, such as the WAIS-IV (*r* = 0.52), the Raven’s APM (*r* = 0.53), and the Advanced Numerical Reasoning Appraisal (*r* = 0.68). Tests of criterion validity compared scores on the assessment to both academic and job performance. The GPAs of nursing students (*r* = 0.30) and the exam scores from an educational psychology class (*r* = 0.42–0.57) were moderately related to scores on the assessment. Scores on the W-GII were also moderately related to supervisor ratings of job performance in numerous industries (*r* = 0.28) and a government agency (*r* = 0.39). This assessment was one of a few assessments that established a relationship between scores on the assessment and everyday behavior outside of a classroom (e.g., job performance).

## 5. Conclusions

It is clear from the review of these assessments that a test developer’s definition of critical thinking impacts the skills or traits that are measured. Still, many of the critical thinking skills assessments measure the same skills (e.g., argument analysis, inductive and deductive reasoning, quantitative reasoning), so there appears to be some overlap in the subscales measured by these assessments. The same cannot be said of critical thinking disposition assessments, where the subscales measured by the assessments vary widely. As the disposition to use one’s critical thinking skills is paramount, this may be a fruitful area for future research. Additionally, many of the critical thinking assessments use realistic scenarios from everyday life, but more work needs to be done to demonstrate scores on these assessments; both the skills and the disposition to use them predict actual behavior.

The previous section began by encouraging educators to overcome the practical challenges associated with critical thinking assessment and by asking colleges and universities to prioritize this important student learning outcome by allotting resources to its assessment and creating a space for educators to discuss ways to improve critical thinking in their classrooms. Educators might find Halpern’s (1998) model for teaching critical thinking useful in this endeavor. The model urges educators to explicitly teach critical thinking skills in all classes (e.g., name the skill being taught), encourage and incentivize students to develop their critical thinking disposition, use real everyday examples to make knowledge transfer more likely to occur, and model metacognitive monitoring in class.

It is important that college students gain critical thinking skills and a disposition to use those skills during their time in the academy, but it is equally important that those who are not fortunate enough to receive a quality higher education learn critical thinking skills and dispositions. Beyond the ivory tower of higher education, there are few opportunities for people to learn and receive feedback about their critical thinking skills. Readers are encouraged to be creative and consider ways to remedy this (e.g., developing short online tutorials or a critical thinking pop-up that would appear on questionable webpages to remind readers to consider the evidence behind the claims). We built an educational game that taught students scientific reasoning (Forsyth et al. 2012; Halpern et al. 2012), why not build one to teach critical thinking and make it accessible to the masses for free?

Even if we were only successful at teaching one critical thinking skill, it could have a major impact on the world. Lilienfeld et al. (2009), argue that if we could overcome confirmation bias, we could have world peace by reducing ideological extremism and intergroup conflict. In a world that is experiencing a war in Israel and political extremism in the United States, that sounds great to me.

## Figures and Tables

**Table 1:** Critical thinking assessment characteristics.

	CCTDI ^a^	CCTST ^b^	CCTT ^c^	CM3 ^d^	E-W ^e^	HCTA ^f^	TER ^g^	W-GII ^h^
Construct	Disposition	Skills	Skills	Disposition	Skills	Skills	Skills	Skills
Respondent Age	18+	18+	10+	5+	12+	18+	Late childhood to adulthood	18+
Format(s)	Digital and paper	Digital	Paper	Digital and paper	paper	Digital	Digital and paper	Digital
Length	75 items	40	52–76 items	25 items	1 problem	20–40 items	35 items	40 items
Administration Time	30 min	55 min	50 min	20 min	40 min	20–45 min	45 min	30 min
Response Format	Multiple-choice	Multiple-choice	Multiple-choice	Multiple-choice	Essay	Multiple-choice and short-answer	Dichotomous choice	Multiple-choice
Fee	yes	yes	yes	yes	no	yes	yes	yes
Evidence—Reliability	yes	yes	yes	yes	no	yes	yes	yes
Evidence—validity	no	yes	no	yes	yes	yes	None available	yes
Credential required for administration	yes	no	no	no	no	no	Developer scores	no

^a^ CCTDI = California Critical Thinking Dispositions Inventory; ^b^ CCTST = California Critical Thinking Skills Test; ^c^ CCTT = Cornell Critical Thinking Test; ^d^ CM3 = California Measure of Mental Motivation; ^e^ E-W = Ennis–Weir Critical Thinking Essay Test; ^f^ HCTA = Halpern Critical Thinking Assessment; ^g^ TER = Test of Everyday Reasoning; ^h^ W-GII = Watson-Glasser Critical Thinking Appraisal II.

## Data Availability

No new data were created or analyzed in this study. Data sharing is not applicable to this article.
